# Radiographic Examination before Dental Extraction from Dentists' Perspective

**DOI:** 10.1155/2023/4970981

**Published:** 2023-03-22

**Authors:** Atheer Talib Jiboon, Faaiz Y. Alhamdani, Nagham Hussein Ali

**Affiliations:** ^1^Department of Oral and Maxillofacial Surgery, College of Dentistry, Mustansiriyah University, Baghdad, Iraq; ^2^Clinical Sciences Department, College of Dentistry, Ibn Sina University of Medical and Pharmaceutical Sciences, Baghdad, Iraq

## Abstract

**Background:**

It is generally agreed that radiographic examination is important before dental extraction. It provides information about the roots and the surrounding tissues. In terms of practice, it does not seem to be a universally implemented protocol regarding the use of dental radiology before dental extraction. Besides, the type of radiographic technique is not specified. Some references prefer periapical dental radiographs. Others prefer orthopantomography), or even cone beam computed tomography Delpachitra et al. (2021) [1]. In terms of the dental practice, it is not clear whether there is a universally adopted protocol regarding the use of dental radiographs before dental extraction. *Aim of the study*. To assess dental professionals' perspective toward radiographic examination before conventional dental extraction.

**Materials and Methods:**

A Google form questionnaire was circulated to different dental professionals using mainly ResearchGate, in addition to different social media platforms.

**Results:**

One hundred and forty-five dentists participated in the questionnaire. The respondents were divided according to the country of current practice: national (Iraqi), regional (Middle Eastern), and international participants. Out of 144 respondents, 51.4% percent of the participants were international, while 40.3% were Iraqis, and 8.3% were from the Middle East. The need for dental radiography in all dental extraction procedures was reported in the majority of responses (*n* = 86). Only 11 dentists think there is no necessity for radiographic examination before conventional extraction. The chi-square test showed a highly significant relationship between the country of current practice and the need for X-ray examination for conventional dental extraction (*P* < 0.01). Seventy-six dentists prefer periapical radiographs. Thirty-five preferred orthopantomography. A highly significant relationship was found between the country of practice and the X-ray technique (*P* < 0.01).

**Conclusion:**

The study showed that there is no universally adopted protocol regarding the use of dental radiography before dental extraction. The country of practice appears to govern the dentists' decisions regarding the need for an X-ray and the type of radiography prior to dental extraction. Periapical radiographs for posterior teeth seem to be the preferable choice before dental extraction.

## 1. Introduction

It is generally agreed that radiographic examination is important before dental extraction. It provides information about the roots and the surrounding tissues.

In terms of practice, it does not seem to be a universally implemented protocol regarding the use of dental radiology before dental extraction. Besides, the type of radiographic technique is not specified. Some references prefer periapical dental radiographs. Others prefer orthopantomography), or even cone beam computed tomography [1]. In terms of the dental practice, it is not clear whether there is a universally adopted protocol regarding the use of dental radiographs before dental extraction.

The current dental practice has shown considerable improvement in terms of diagnosis and management. The utilization of a diversity of dental educational materials in social media [2] and telemedicine seems to play a significant role [3]. However, oral hygiene measures remain suboptimal worldwide [4, 5]. Advanced tooth decay still represents a problem. Hence, dental extraction remains an accepted treatment. Dental extraction continues to be the most common oral surgical procedure in dental practice [6].

Doing proper dental extraction is an essential prerequisite for a qualified dentist. Proper dental extraction mandates performing the procedure with the least possible trauma to the surrounding tissue [7]. Minimizing trauma to the adjacent tissue during dental extraction necessitates adequate knowledge about the condition of the tooth and the surrounding structures.

It is generally agreed that radiographic examination is important before dental extraction [8]. It is essential in providing information about different root conditions and abnormalities, such as resorption, ankylosis, and hypercementosis. In addition, it shows the presence of any tooth-related pathologies and the proximity of vital structures and surrounding structures [9, 10].

In terms of practice, it does not seem to be a widely implemented protocol regarding the use of dental radiology before dental treatment. Besides, there is no specified radiographic technique for each condition. Some references refer to dental radiograph [11]. Others prefer orthopantomographic view [12] or even cone beam computed tomography [13, 14]. In Iraqi Dental Iraqi Academic Institutes, dental radiographs are not always requested for dental extraction.

## 2. Aim of the Study

To investigate dental professionals' perspective toward dental radiography before conventional dental extraction.

## 3. Materials and Methods

A Google form questionnaire was circulated to different dental professionals in different countries and diverse academic and professional qualifications. The questionnaire aimed to identify the areas of agreement on the situations where the radiographic investigation might be necessary.

The questionnaire was circulated using ResearchGate and different social media platforms, such as Viber, WhatsApp, and Facebook Messenger. Respondents were divided according to the country of current practice into nationals (Iraqis), Middle Eastern, and internationals.

Statistical analysis was performed using both descriptive and inferential statistical tests. The chi-square test was used to examine the relationship between nominal and ordinal variables. The Spearman correlation test was used to identify the relationship between ordinal variables. *P* < 0.05 was considered the statistical value for significance. IBM® SPSS, Ver 25 was the implemented statistical software for analysis.

## 4. Results

### 4.1. Demographic Data

One hundred and forty-five dentists participated in the questionnaire (Table 1). Out of 144 respondents 51.4% percent of the participants were international, while 40.3% were Iraqis, and 8.3% were from the Middle East. The international dentists were: 34 from the United Kingdom, 9 from Canada, 7 from the United States of America, 5 from Sweden, 3 from Brazil, 3 from India, 2 from Australia, 2 from Malaysia, 2 from Tanzania, 1 from each of the following countries; Hungary, Portuguese, Nigeria, New Zealand, Italy, and South Korea. Out of 12 Middle Eastern countries, 5 were from United Arab Emirates, 2 from each of the following countries; Oman, Jordan, and Palestine. One participant was from Turkey.

### 4.2. Medico-Legal Obligation

**Figure 1:**
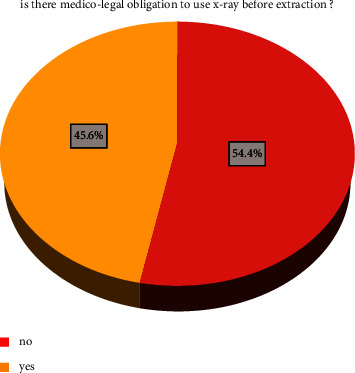
Is there medico-legal obligation to use X-ray before extraction?

As shown in Figure 1, the majority of respondents stated that there is no medico-legal obligation regarding taking an X-ray before dental extraction. Seven dentists out of 58 (12.1%) who practice dentistry in Iraq reported the presence of medico-legal obligation in using radiographic investigation before dental extraction. Six dentists out of 12 (50%) who practice dentistry in the Middle East reported the presence of a medico-legal obligation of using X-ray, whereas 55 out of 74 dentists (74.3%) with current international practice reported the presence of such an obligation.

### 4.3. The Need for Dental Investigation

**Figure 2:**
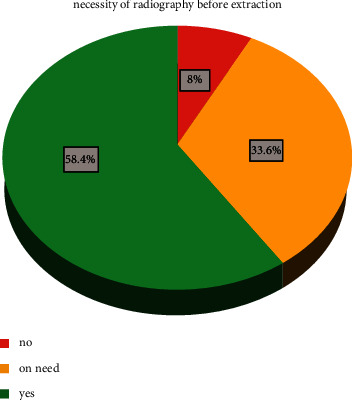
Necessity of radiography before extraction.

From dentists' perspectives, the need for dental radiography in all dental extraction procedures was reported in the majority of responses (*n* = 86, 59%). Dentists who think radiography is useful in certain situations were 48 (33%) dentists. Only 11 (8%) dentists think there is no necessity for radiographic examination before conventional extraction (Figure 2). The chi-square test showed a highly significant relationship between the country of current practice and the country of highest academic qualification and the need for X-ray examination for conventional dental extraction (*P* < 0.01).

Most of the dentists (*n* = 47, 54.7%), who believe that X-ray is necessary before conventional extraction chose PA radiography as their preferred method. Twenty-two (25.6%) chose both PAs in conjunction with OPG. Fifteen dentists (17.4%) preferred orthopantomography, whereas only 2 participants (2.3%) chose cone beam computed tomography as their preferred diagnostic modality.

It should be mentioned, however, that in foreign countries, there was no significant relationship between the qualification and the need for radiographic examination before the extraction process (*P* > 0.05).

### 4.4. Types of Dental Radiography before Dental Extraction

**Figure 3:**
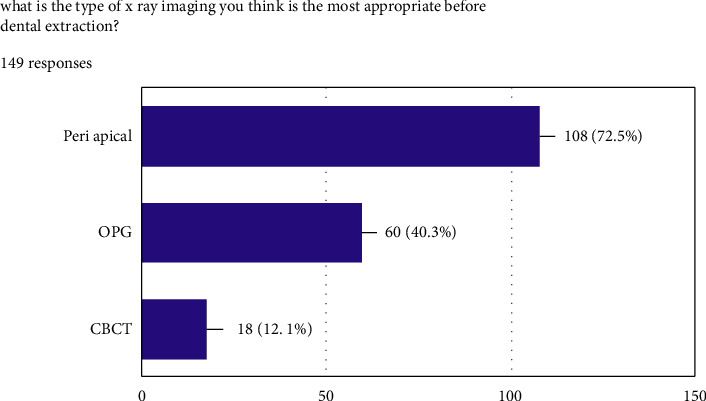
What is the type of X-ray imaging you think is the most appropriate before dental extraction?

Figure 3 gives the percentages of each X-ray technique preferred by the respondents. Seventy-six dentists prefer periapical radiographs. Thirty-five preferred orthopantomography. More than one type of X-ray was preferred by 30 participants, whereas 4 dentists preferred cone beam computed tomography. A highly significant relationship was found between the country of practice and the X-ray technique (*P* < 0.01), but no significant relationship was found between the academic qualification and the radiographic technique (*P* > 0.05). Neither academic qualification, nor years of experience were found to have a significant relationship with the type of radiograph (*P* > 0.05).

**Figure 4:**
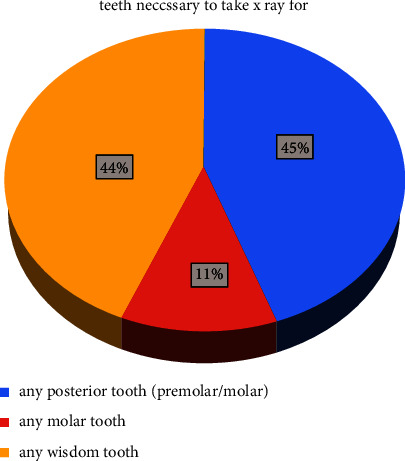
Teeth necessary to take X-ray for.

The choice of teeth for radiographic investigation is demonstrated in Figure 4. All posterior teeth, including premolars and all wisdom teeth categories showed comparable preferences (no = 54 and 53, respectively). Only 14 dentists prefer radiographic examination for molar teeth. The country of practice was found to have a highly significant relationship with the type of tooth to be examined before extraction (*P* < 0.01).

### 4.5. Justifications for Radiographic Examination

**Figure 5:**
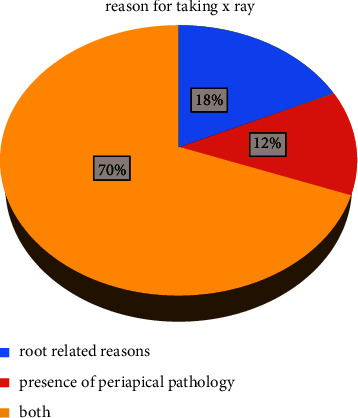
Reasons for taking X-ray.

As shown in Figure 5, almost 2/3 of the respondents (73 dentists) stated their reason for taking an X-ray for the examination of both tooth roots and the presence of periapical pathology. This is followed by 19 dentists who justified their request for X-rays by root-related reasons alone. Only 11 out of 105 dentists stated that the presence of a periapical lesion is the reason for an X-ray examination. Country of practice again was found to have a highly significant relationship with the reason for taking radiographs before extraction (*P* < 0.01). Country of current practice and years of experience were not found to have a significant relationship with the reason for radiographic examination (*P* > 0.05).

## 5. Discussion

The dentist has the responsibility of dental radiographic imaging, interpretation, and making the clinical imaging accordingly. This means, by default radiographic examination and interpretation is an essential part in the dentist's job [15]. This makes radiographic examination an important diagnostic tool in the dentist's arsenal to reach the accurate diagnosis. However, according to both the American Dental Association and the Faculty of General Dental Practice in the United Kingdom, there is no evidence to support routine radiographic investigation before dental extraction, and it is governed by the dentist's judgment. [16, 17] In Iraqi dental schools, radiographic investigation is not an essential requirement before dental extraction [18].

The country of practice, as revealed by the study data, seems to be a major influential factor in the need for radiography before exodontia, whatever the dentist's qualification was. This might reflect the importance of medico-legal obligations in certain countries. The need for a radiograph before each dental extraction will not only protect the dentist from any error in decision-making but also reflects the importance of shared decision-making. The patients should be informed about the treatment option in the light of the diagnosis and allowed to be involved in the treatment decision [19]. This protects the dentist from any claim of negligence [20].

Medico-legal obligations, as stated in the literature, do not specify the necessity of dental radiography before dental extraction. They rather stress the negligence aspect of practice, which might result in various complications [21]. These complications might the dentist's position against any medico-legal claims by the patient [20]. Medico-legal obligations, as the study suggests, do not enjoy the same level of awareness and implementation among different countries.

It is worth mentioning that dental practitioners should consider the radiation hazard if a radiographic examination is to be considered. Luckily, the introduction of digital periapical radiography seems to offer less radiation hazard compared to the conventional periapical film [22]. This could encourage dentists who work in well-equipped dental centers to use digital periapical radiographs in their practice.

Over half of the respondents preferred periapical radiographs before dental extraction. Understandably, periapical radiography provides more accurate information about the tooth condition compared to orthopantomography. Periapical radiographs are the gold standard two-dimensional imaging method. It offers adequate information about the roots and associated pathologies [23, 24]. Cone beam computed tomography was preferred by a small minority of the participants, despite its superiority [25]. This radiographic modality is more expensive and not available in most private dental practice premises.

Orthopantomography has been chosen by almost half of the respondents, either alone or in conjunction with periapical radiographs. Given the limited view provided by periapical radiographs, the orthopantomography has the advantage of providing an overview of dental arches and jawbones. Besides, it is a useful alternative imaging technique for patients with severe gag reflexes [24, 26]. However, it has a major drawback, which is the superimposition at the premolar area. This technique-related superimposition affects the clarity of the image in this particular region [27].

The country of current practice influences the choice of teeth to be examined radiographically. According to the study data, molars do not have a particular radiographical feature compared to premolars. Both molars and premolars need to be examined for their roots and their relationship with the adjacent vital structures, such as the maxillary sinus [28–30] and the inferior alveolar canal, especially for impacted lower third molars [31, 32].

Neither qualification nor years of experience seem to influence the dentist's decision regarding the choice of X-ray type and what is it needed for. This might reflect that radiographic investigation is a matter of regulation rather than opinion. Whether this opinion is influenced by the qualification or the experience is debatable.

An important limitation of the study is the number of respondents. Wider participation would provide better insight into the differences in practice regarding radiographic examination before dental extraction.

Nevertheless, the dentists' responses could help to establish a guideline for academic Iraqi dental institutes. These guidelines can be implemented in both the public and private sectors.

## 6. Conclusion

The study showed that there is no universally adopted protocol regarding the use of dental radiography before dental extraction. The country of practice appears to govern the dentists' decisions regarding the need for an X-ray and the type of radiography prior to dental extraction. Periapical radiographs for posterior teeth seem to be the preferable choice before dental extraction.

## Figures and Tables

**Table 1:** Study demographics.

Study variables	No. of respondents
*Country of current practice*	
Iraq	58
Regional (Middle East)	12
International	74

*Years of practice*	
Less than 5 years	17
5 to 10 years	27
11 to 15 years	25
16 to 20 years	26
More than 20 years	50

*Country of the highest degree of graduation*	
Iraq	91
Regional (Middle East)	4
International	50

*Qualification*	
Bachelor degree	53
Diploma/Master	64
PhD/Doctorate	25

## Data Availability

Data are available upon request.
